# Cachexia Anorexia Syndrome and Associated Metabolic Dysfunction in Peritoneal Metastasis

**DOI:** 10.3390/ijms20215444

**Published:** 2019-10-31

**Authors:** Rami Archid, Wiebke Solass, Clemens Tempfer, Alfred Königsrainer, Michael Adolph, Marc A. Reymond, Robert B. Wilson

**Affiliations:** 1 Department of General & Transplant Surgery, University Hospital Tübingen, D-72076 Tübingen, Germany; alfred.koenigsrainer@med.uni-tuebingen.de (A.K.); marc.reymond@med.unit-tuebingen.de (M.A.R.); 2 National Center for Pleura and Peritoneum, University Hospital Tübingen, D-72076 Tübingen, Germany; Wiebke.solass@med.uni-tuebingen.de; 3 Institute of Pathology and Neuropathology, University Hospital Tübingen, D-72076 Tübingen, Germany; 4 Department of Gynecology and Obstetrics, Ruhr-University Bochum, 44625 Herne, Germany; clemens.tempfer@marienhospital-herne.de; 5 Nutrition Unit, Department of Anesthesiology, University Hospital Tübingen, D-72076 Tübingen, Germany; micahel.adolf@med.uni-tuebingen.de; 6 Department of Upper Gastrointestinal Surgery, UNSW, Liverpool Hospital, Sydney, NSW 2170, Australia; robert.wilson@unsw.edu.au

**Keywords:** cancer cachexia, cachexia anorexia syndrome, peritoneal metastasis, cancer metabolism

## Abstract

Patients with peritoneal metastasis (PM) of gastrointestinal and gynecological origin present with a nutritional deficit characterized by increased resting energy expenditure (REE), loss of muscle mass, and protein catabolism. Progression of peritoneal metastasis, as with other advanced malignancies, is associated with cancer cachexia anorexia syndrome (CAS), involving poor appetite (anorexia), involuntary weight loss, and chronic inflammation. Eventual causes of mortality include dysfunctional metabolism and energy store exhaustion. Etiology of CAS in PM patients is multifactorial including tumor growth, host response, cytokine release, systemic inflammation, proteolysis, lipolysis, malignant small bowel obstruction, ascites, and gastrointestinal side effects of drug therapy (chemotherapy, opioids). Metabolic changes of CAS in PM relate more to a systemic inflammatory response than an adaptation to starvation. Metabolic reprogramming is required for cancer cells shed into the peritoneal cavity to resist anoikis (i.e., programmed cell death). Profound changes in hexokinase metabolism are needed to compensate ineffective oxidative phosphorylation in mitochondria. During the development of PM, hypoxia inducible factor-1α (HIF-1α) plays a key role in activating both aerobic and anaerobic glycolysis, increasing the uptake of glucose, lipid, and glutamine into cancer cells. HIF-1α upregulates hexokinase II, phosphoglycerate kinase 1 (PGK1), pyruvate dehydrogenase kinase (PDK), pyruvate kinase muscle isoenzyme 2 (PKM2), lactate dehydrogenase (LDH) and glucose transporters (GLUT) and promotes cytoplasmic glycolysis. HIF-1α also stimulates the utilization of glutamine and fatty acids as alternative energy substrates. Cancer cells in the peritoneal cavity interact with cancer-associated fibroblasts and adipocytes to meet metabolic demands and incorporate autophagy products for growth. Therapy of CAS in PM is challenging. Optimal nutritional intake alone including total parenteral nutrition is unable to reverse CAS. Pressurized intraperitoneal aerosol chemotherapy (PIPAC) stabilized nutritional status in a significant proportion of PM patients. Agents targeting the mechanisms of CAS are under development.

## 1. Introduction

Cachexia anorexia syndrome (CAS), defined by an ongoing loss of skeletal muscle mass (with or without loss of fat mass), is a common feature of advanced malignancy and some palliative medical conditions. CAS is a leading cause of reduced quality of life and of mortality in cancer patients [1,2]. Diagnostic criteria of CAS include weight loss greater than 5%, or weight loss greater than 2% in individuals already showing depletion, according to current bodyweight and height (body mass index (BMI) <20 kg/m^2^) or skeletal muscle mass (sarcopenia) [2]. 

The nature of CAS is multifactorial [2] and includes involuntary weight loss, reduced food intake, increased energy consumption, and protein and fat catabolism. Loss of appetite (anorexia) is common among patients with advanced cancer, with a reported prevalence as high as 66% [3,4,5]. Anorexia is caused by ghrelin resistance, cytokine release, and a decreased hypothalamic drive to eat, but also by pain, weakness, dry mouth, difficulty chewing or swallowing, dysphagia, constipation, chemosensory disturbances (e.g., taste and smell), early satiety, and nausea. These can all contribute to reduced caloric intake [2,4,5,6].

Peritoneal metastasis (PM), defined as the transcoelomic spread of cancer onto the visceral and parietal surfaces of the peritoneum, is a frequent condition in gastrointestinal and gynecological cancers [7]. PM causes considerable morbidity and mortality, despite recent improvements in multimodal therapy [8]. In patients with PM, intestinal dysfunction and decreased gastrointestinal motility caused by tumor infiltration of the bowel contribute to anorexia [9]. Anorexia and nausea is also exacerbated by medications, in particular systemic chemotherapy and morphine derivatives [10]. Malignant ascites is common in PM patients. Repeated drainage of ascites improves symptoms such as abdominal fullness and breathlessness, but worsens protein loss, sarcopenia, and renal dysfunction [11].

Despite its essential contribution to the pathogenesis of cancer CAS, reduced caloric intake cannot entirely explain the metabolic catabolism observed in this condition. Optimal compensated nutritional intake including total parenteral nutrition (TPN) is unable to curb the progression of cachexia and protein loss in cancer patients, as compared to simple starvation [12,13].

## 2. Physiology of Starvation

**Figure 1:**
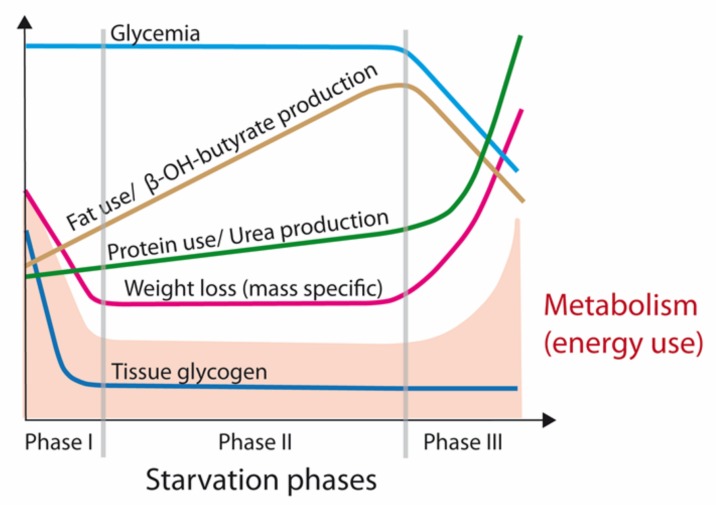
Phases of starvation. In the first phase of starvation, glycogen stores are utilized rapidly and the body reacts by reducing metabolic needs and resting energy expenditure (REE). In Phase II, gluconeogenesis is the main energy provider, initially from protein carbon chains, then increasingly from fatty tissue. In the third phase, fat stores are depleted. Resting energy metabolism and protein use greatly increase, leading to end of life by exhaustion (adapted from [15]).

An examination of the physiology of starvation is relevant to an understanding of the syndrome of cancer CAS and potential treatments. As shown in Figure 1, three fasting/starvation phases can be defined based on a particular pattern of body mass loss and the substrates being metabolized [14,15]. Phase I is short, ranging from many hours to a week, with increasing reliance on body stores of glycogen, lipids, and protein as well as progressive depletion of glycogen stores to maintain glucose availability. Within 24 h, glycogen stores of the body, especially in the liver, are utilized by up to 85% to provide energy. Glycogen metabolism is replaced by gluconeogenesis (GNG), where required carbon chains are mainly supplied by muscle proteins [15,16]. Then, progressively, metabolic activity is slowed, together with protein utilization [14,15]. The human body adapts its consumption of energy reserves during the decreased caloric intake and fat free mass (FFM) of starvation by decreasing resting energy expenditure (REE). 

Under physiological conditions, protein mass is preserved with a progressive selection of fat as a body fuel including glycerol for GNG. Continued important sparing of visceral and muscle proteins is characteristic of phase II of starvation, lasting several weeks. Although amino acids are still utilized for continued gluconeogenesis, there is an increase in the production of lipid-derived ketone bodies that serve as a glucose substitute for ensuring organ function [14,15].

However, prolonged fasting eventually results in the depletion of fat stores and, since downregulation of fatty acid oxidation is not indefinite, the contribution of amino acids to fuel metabolism by gluconeogenesis increases again. This switch is observed in phase III of starvation, and announces the last few weeks (or days) of life [15,17,18]. In extremely malnourished persons near death (BMI < 10 kg/m^2^), there is a paradoxical increase in REE, which is known as ‘King Penguin’ syndrome. The increased REE in the final stage of starvation is associated with elevated protein catabolism, increased urinary nitrogen excretion, near zero fat mass, and low fatty acid levels. This paradoxical response is related to the mobilization of the last available muscle mass (including visceral/cardiac/diaphragm/respiratory). The energy production from protein has a decreased metabolic efficiency [16]; energy per wet mass of protein being eight times lower than fat. Modifications of cellular and mitochondrial membranes also contribute to the increased REE, explaining the accelerated loss of body mass during phase III [15].

## 3. Cancer Cachexia Anorexia Syndrome (CAS)

CAS should be distinguished from starvation (Table 1). Preserved appetite, ghrelin response, sparing of resources, and decreased energy consumption is an essential adaptation of animals and humans during prolonged fasting. However, ghrelin resistance, tumor and host derived cytokine release, loss of appetite, metabolic inefficiency, protein loss, and energy wasting are characteristic of CAS [12,19].

Cancer cachexia is characterized by cancers recruiting a catabolic host response to supply nutrients for anabolic tumor metabolism and cancer progression. This results in a negative energy balance in the host. The energy consumption of a tumor can be estimated based on REE, glucose turnover, glucose recycling, and oxygen consumption in cancer patients. This tumor energy consumption varies widely from 190–470 kcal/kg tumor/day. Depending on the tumor burden and tumor metabolic activity, the overall tumor energy cost to the host can range from 100–1400 kcal/day [20]. The impact of this tumor energy cost on the patient is dependent on patient body mass. For example, tumor energy costs of 300 kcal/day will be 25% of REE of a patient at a normal REE of 1200 kcal/day, but only 15% of REE for a patient with a normal REE of 2000 kcal/day. This energy consumption by cancers and elevated REE contributes substantially to the progressive cachexia and energy store depletion in cancer CAS patients [20].

Elevated REE is observed particularly in patients with metastatic cancer or long duration of disease [3,21], as confirmed in our cohort of patients with peritoneal metastasis [3]. At first hospital admission, malnutrition was moderate with a stage B subjective global assessment score (SGA). Body mass index was initially conserved, as were the skeletal muscle percentage, the visceral fat level, and body fat percentage. These were measured by bioelectrical impedance analysis (BIA) and skinfold thickness. Total serum protein and serum albumin were at the lower limit of normal. Later in the course of disease, nutritional status worsened, C-reactive protein (CRP) increased, skeletal muscle percentage, albumin and total protein serum levels further deteriorated, and REE increased until death. In parallel, the ascites volume also increased substantially, causing further protein loss and severe hypoalbuminemia [3]. These effects were more pronounced in patients with PM of gastrointestinal origin than in women with PM of ovarian origin [3]. Presence of CAS in PM patients was confirmed in a later study of 84 women with PM derived from gynecological cancers. At first hospital admission, mean visceral fat level, skeletal muscle mass, albumin and protein levels were below normal limits and diagnostic criteria of CAS were met in 23% of patients [22]. 

## 4. Metabolic Dysfunction in Peritoneal Metastasis

Millions of cancer cells may remain within the peritoneal cavity after a cancer resection operation [23]. Fortunately, most of them do not give rise to PM. Cancer cells shed into the peritoneal cavity have lost contact with their tumor microenvironment. They are hypoxic (in the absence of vascular supply) and have less access to nutrients. Detachment of cells from the extracellular matrix into the coelom initiates a cascade of metabolic adaptations. In order to survive, these cells have to find a metastatic niche (e.g., a peritoneal wound after surgery) or to develop resistance to anoikis (i.e., evasion of programmed cell death). Resistance to anoikis requires a switch from oxidative phosphorylation (OXPHOS) to cytosolic glycolysis, which is mediated by glycolytic enzymes and hypoxia inducible factor-1α (HIF-1α) [24]. 

Glucose is a key energy substrate of cells used to generate Adenosine triphosphate (ATP), maintain redox state, and create biomass. In normal eukaryotic cells as well as in cancer cells, glucose can be catabolized in the cytoplasm via glycolysis to lactate, or alternatively to carbon dioxide and water in the mitochondria via OXPHOS [25]. 

Under normal physiological conditions, there is minimal lactate production and most cells produce ATP via OXPHOS. However, during strenuous exercise or hypoxia, lactate production is greatly increased and lactate is either recycled locally or transported to the liver for recycling by gluconeogenesis and lactate dehydrogenase (LDH) via the Cori cycle. This is extremely energy inefficient, as six ATP are consumed to produce one molecule of glucose. In advanced malignancies, hepatic GNG is markedly increased, and cancer derived lactate is regenerated to glucose, which is scavenged by cancers for further proliferation and anabolism. The liver also contributes to cancer cachexia by recycling proteins from muscle wasting for amino acid production. The resulting alanine and glutamine provide energy substrates for GNG and tumor growth. Insulin resistance and decreased circulating levels of IGF-1 are also associated with the increased hepatic gluconeogenesis in CAS, which contributes to muscle wastage by diminishing muscle utilization of glucose and inhibiting muscle growth [26,27].

A significant increase in energy exploitation by cancers can be explained by the ‘Warburg effect’ [28]. The Warburg effect refers to Otto Warburg’s original observation that cancers preferentially use ‘aerobic’ cytosolic glycolysis as a source of energy rather than the more efficient mitochondrial pathway of OXPHOS, even under normoxic conditions [24,25]. Aerobic glycolysis is 90% less efficient, but at the same time 10–100 times more rapid in producing ATP when compared to mitochondrial OXPHOS (2 ATP vs. 30 ATP per glucose molecule) [20]. Thus, a major upregulation of glucose transporters and glucose usage, glycolytic enzymes, and lactate production is required for the maintenance of ATP production to ensure tumor cell survival [24,29]. Variations of the glycolytic ratio within tumors (from 0 to 100%) can alter the energy cost of tumors over a 2- to 3-fold range [20].

In the microenvironment of solid tumors, HIF-1α plays a key role in activating both aerobic and anaerobic glycolysis, and increasing metabolic flux of glucose, lipid, and glutamine in cancer cells [24,30]. Cytoplasmic glycolysis results from HIF-1α-induced upregulation of hexokinase II, phosphoglycerate kinase 1 (PGK1), pyruvate dehydrogenase kinase (PDK), pyruvate kinase muscle isoenzyme 2 (PKM2), lactate dehydrogenase (LDH), and glucose transporters (GLUT1,2) [24,31,32]. Feeding glucose into the hexokinase pathway to produce lactate, and into the pentose phosphate pathway (PPP) for the production of Nicotinamide adenine dinucleotide phosphate (NADPH) and glutathione, rather than the production of acetyl-CoA from pyruvate for mitochondrial OXPHOS, helps to minimize electron leakage from the respiratory chain in dysfunctional mitochondria during hypoxia. This protects against the generation of reactive oxygen species (ROS) and oxidative stress in hypoxic or ‘homeless’ cells and prevents mitochondrial mediated apoptosis. At the same time, HIF-1α can stimulate the utilization of glutamine and fatty acids as alternative substrates to glucose for mitochondrial energy production [24].

## 5. Reverse Warburg Effect

**Figure 2:**
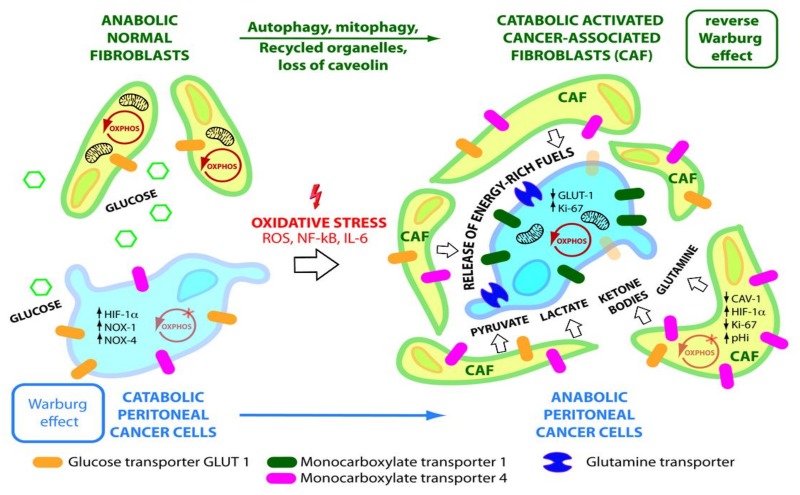
Reverse Warburg Effect. Normal fibroblasts/mesothelial cells are transformed into glycolytic cancer-associated fibroblasts (CAFs) under the influence of hypoxic cancer cells. In the process, fibroblasts lose their mitochondrial function via cytokines, oxidative stress, and mitophagy and come to rely on cytoplasmic glycolysis. CAFs release energy substrates (pyruvate, lactate, ketone bodies, glutamine) into the tumor microenvironment (TME) via autophagy and MCT4. These are taken up by MCT1 and glutamine transporters in anabolic cancer cells to maintain OXPHOS and mitochondrial ATP production (adapted from [33,34]).

The use of glycolysis by hypoxic cancer cells is explained by the inhibition of mitochondrial pyruvate dehydrogenase (PDH) by PDK, and their inability to process glucose via OXPHOS. However, the majority of aerobic cancer cells contain functional mitochondria [35]. As well as a glycolytic phenotype, OXPHOS is maintained within cancer tissue [35]. These observations support a further theory named the ‘reverse Warburg effect’ [36]. The metabolic reprogramming in cancer is explained in this theory on the basis of a two-compartment model with interactions between catabolic cancer-associated fibroblasts (CAFs) and anabolic cancer cells to meet metabolic demands and maintain ATP production in cancer cells [25,33]. The transformation of normal fibroblasts into activated CAFs is modulated by ROS and IL-6. Reactive oxygen species produced by catabolic cancer cells and the downregulation of the Krebs cycle enzyme isocitrate dehydrogenase 3α (IDH-3α) upregulates HIF-1α and NADH dehydrogenase (ubiquinone) 1 alpha subcomplex, 4-like 2 (NDUFA4L2), which in turn inhibits OXPHOS and promotes CAF glycolysis [33] (Figure 2, 4).

Furthermore, downregulation of stromal Cav-1 plays an important role in CAF transformation and additionally induces a self-destructive autophagy/mitophagy program in CAFs to create a nutrient-rich tumor microenvironment [36,37,38]. CAFs in turn fuel cancer cells by producing and exporting high-energy metabolites, especially lactate, pyruvate, glutamine, and ketone bodies. Catabolite transporters allow for export from CAFs (monocarboxylate transporter 4 (MCT-4)) and uptake (monocarboxylate transporter 1 (MCT-1), glutamine transporter) into adjacent cancer cells, where they are used to generate energy via OXPHOS or for anabolic processes to support cell division and tumor mass [33,39]. The export of lactic acid into the extracellular space by CAFs and uptake of the lactate by adjacent cancer cells also maintains an acidic extracellular tumor microenvironment (pHe) and alkaline intracellular CAF environment (pHi). This enables tumor cell intravasation, immune evasion, angiogenesis, and chemotherapy drug resistance and is fundamental to the survival and progression of PM [29] (Figure 2).

The metabolic transformation of normal fibroblasts into CAFs is associated with the overexpression of enzymes involved in glucose uptake (GLUT1) and in cytoplasmic glycolysis such as HK2, 6-phosphofructokinase liver type (PFKL), and PKM2 [33,40]. In the tumor mass, CAFs have the largest increase in glucose uptake. Thus the increased 2-deoxy-2-[^18^F] glucose (^18^F-FDG) uptake in the PET scanning (SUVmax) of solid tumors is mainly due to increased glucose uptake into CAFs as a result of the reverse Warburg effect, rather than increased uptake into cancer cells [34,41] (Figure 2).

## 6. Host Inflammatory Response and CAS

The increased cytoplasmic glycolysis, glucose transport, and glucose levels within the tumor mass, especially under hypoxic conditions, are associated with an increase in reactive aldehydes (e.g., methylglyoxal), non-enzymatic glycation, and oxidation of proteins. The products of this process are advanced glycation end products (AGE), also known as glycotoxins (e.g., hydroimidazolone (MG-H1) and argpyrimidine) [42]. AGEs have numerous metabolic and signaling effects on the host and on tumor progression. They act through interaction with their AGE-Receptor (RAGE) [42,43]. RAGE is a transmembrane multi-ligand protein receptor. AGE-RAGE interaction activates intracellular downstream signaling including nuclear factor-κB (NF-κB), HIF-1α, ERK, and AKT pathways involved in inflammation, cell proliferation, autophagy, carcinogenesis, muscle wasting, and protection against oxidative stress [42,44]. AGE-RAGE ligand binding increases levels of the pro-inflammatory (M1) macrophage markers, iNOS and CD86, and the pro-inflammatory cytokines, IL-6 and TNF-α [45]. RAGE is also a receptor for damage associated molecular pattern (DAMP) molecules, which originate from damaged cells and alert the immune system to tissue trauma [46]. This also promotes the systemic inflammatory response in the host. For example, high-mobility group box 1 (HMGB1) is a nuclear protein released by necrotic cells, NK cells, macrophages, and dendritic cells. HMGB1 is a DAMP that acts as a chemokine and a cytokine in the extracellular compartment by binding to RAGE and activating NF-κB pathways, thereby inducing the release of cytokines such as IL-1, IL-6, and TNF-α and contributing to muscle proteolysis and autophagy [45].

Glycolytic enzymes such as PGK1 are involved in the initiation of autophagy in tumor cells under hypoxic conditions with deprivation of resources. Via autophagy, cancer cells are able to recycle damaged or extruded cell organelles such as mitochondria and to utilize them for anabolic processes and/or energy production [47].

The metabolic changes observed in cancer CAS patients are related to an increased systemic inflammatory response, rather than just an adaptation to starvation. Serum CRP is a cytokine that is elevated in patients with PM, particularly in aggressive malignancies such as sarcomatoid malignant peritoneal mesothelioma [48]. Such elevation of CRP is associated with increased REE, protein catabolism, and a higher risk of death [49]. In our cohort of patients with PM, CRP was increased by 6-fold, reflecting major and chronic systemic inflammation [3]. 

Cytokines such as TNF-α, IL-1, IL-6, and interferon-gamma (IFN-γ) are released by both cancer cells and host immune cells (macrophages/lymphocytes). These are involved in mediating the pro-inflammatory state, stress response, anorexia, sickness behavior, hypermetabolism, and accelerated breakdown of protein, muscle, and adipose tissues in cancer cachexia patients [12]. TNF-α (originally called cachexin) activates the ubiquitin-proteasome pathway via pro-inflammatory NF-κB, but also synergizes with IFN-γ and IL-1. These pro-inflammatory cytokines are also transported across the blood–brain barrier. They interact with the luminal surface of the brain endothelial cells, causing release of appetite-suppressing substances [50]. Cytokine receptors are found in the hypothalamic areas of the brain that control food intake, appetite, and hunger [50,51]. For example, in the arcuate nucleus of the hypothalamus, neuropeptide Y/agouti-related peptide neurons promote appetite (orexigenic), respond to ghrelin, are inhibited by Th1 cytokines (TNF-α, IL-6), and activate the parasympathetic nervous system to decrease REE. Conversely, the pro-opiomelanocortin/cocaine and amphetamine regulated transcript neurons promote anorexia (anorexigenic), are inhibited by ghrelin, stimulated by white adipose tissue (WAT) cytokines (e.g., leptin, adiponectin), and activate the sympathetic nervous system to increase REE via browning of AT and the uncoupling protein (UCP) [1,51,52]. However, neutralizing antibody blockade of TNF-α (etanercept/infliximab), IL-6 (clazakizumab), or IL-1 receptors (MABp1) have not been proven to substantially improve CAS in animal or human trials [27]. 

Patients with cancer CAS have been shown to respond poorly to immune checkpoint inhibitors including pembrolizumab, nivolumab, atezolizumab, and ipilimumab. This is related not only to malnutrition and hypoalbuminemia, but also the effect of cytokine release. In cancer cachexia animal models, elevated IL-6 has been shown to promote glucocorticoid-mediated immune suppression, which results in limited T-cell chemotaxis and poor response to immune checkpoint inhibition in the tumor microenvironment. By combining IL-6 inhibition with programmed death ligand-1 (PDL-1) targeted therapy in pancreatic adenocarcinoma, improved overall T-cell activation, and anti-tumor response was achieved in a murine model [53,54]. This is relevant to patients with PM and CAS, as cancer cells in the peritoneum recruit peritoneal mesothelium, fibroblasts, monocytes, adipocytes, and endothelial cells to form a supporting stroma, which enables resistance to anoikis and evasion of T cell immunosurveillance [24,29].

## 7. Catabolism of Fat Tissue

**Figure 3:**
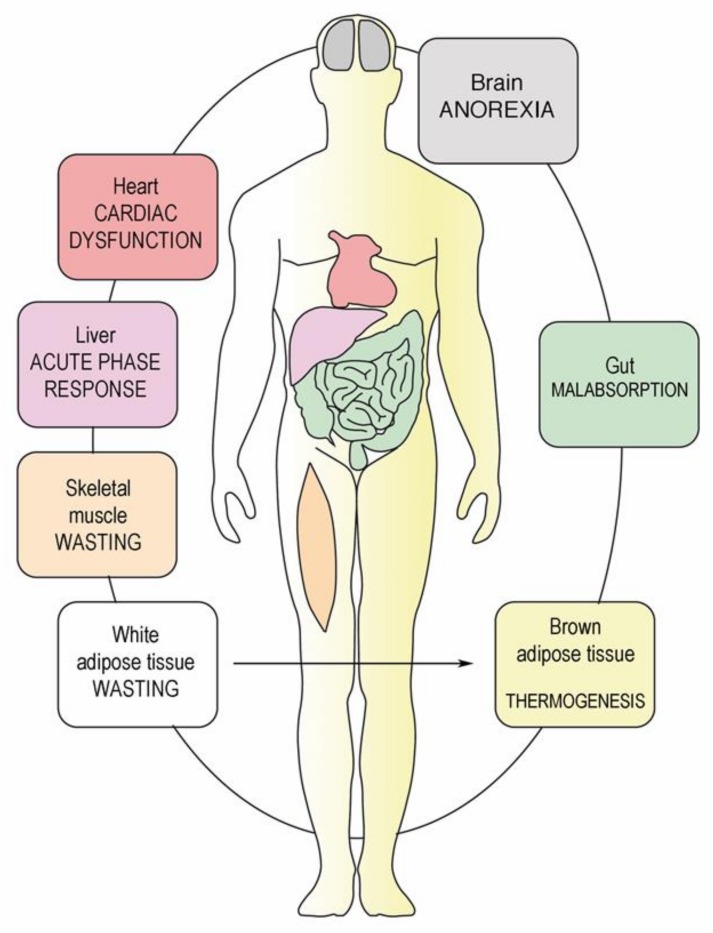
CAS is a multiorgan syndrome. Peritoneal metastasis induces a systemic inflammatory response triggered by hormonal and inflammatory mediators [59]. Changes caused by PM involve not only the intestines, but also brown and white adipose tissue, muscle, brain, liver, and heart. Thus, CAS in PM patients should be considered as a multi-organ, and not an abdominal syndrome (adapted from [12]).

**Figure 4:**
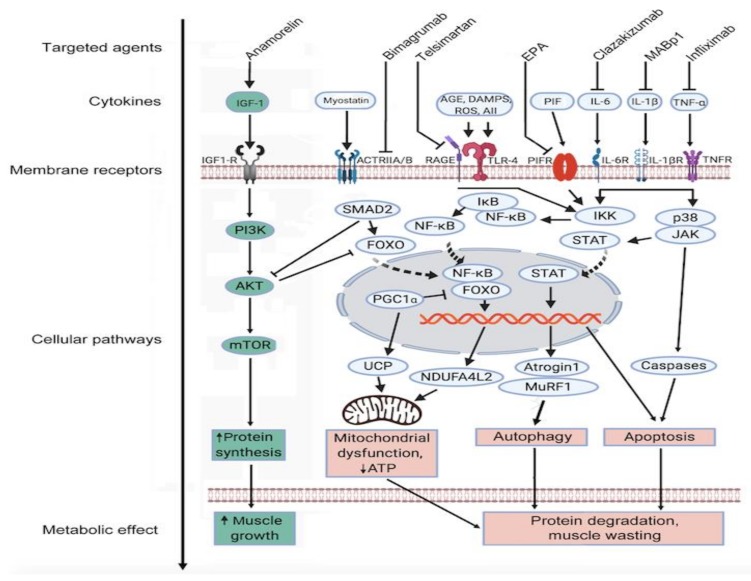
Pathways in muscle catabolism in cancer CAS. Under normal physiological conditions, anabolic (green) and catabolic (red) muscle metabolic signaling pathways are balanced via reciprocal control of the protein kinase AKT, SMAD, and forkhead family transcription factors (FOXO), and muscle mass remains constant. Muscle wasting in cancer CAS is mediated by decreased host insulin-like growth factor-1 (IGF-1) and increased levels of circulating cytokines including prostaglandins, tumor necrosis factor alpha (TNF-α), interleukin-1β (IL-1β), and interleukin-6 (IL-6). The ensuing mitochondrial dysfunction, autophagy, and apoptosis result in muscle degradation and wasting. Whilst several targeting agents are available, their efficacy in cancer CAS remains to be determined. For more details refer to the text (Adapted from Argiles et al. [12]).

The depletion of WAT in both visceral and subcutaneous depots also plays a central role in cancer cachexia [55,56]. Inflammatory cytokines produced by the tumor or adipose tissue such as IL-6, TNF-α, and interleukin-1 beta (IL-1β) contribute to lipolysis, fat oxidation, and decreased lipogenesis as well as browning of white adipose tissue (beige adipose tissue). Differentiation of white adipocytes into brown adipocytes is regulated at a transcriptional level by peroxisome proliferator-activated receptor gamma (PPARγ) and PPARγ coactivator 1-alpha (PGC-1α). This is associated with the upregulation of expression of UCP, increased thermogenesis (non-shivering), loss of ATP production, and catabolic wasting of energy [57]. The two lipases adipose triglyceride lipase (ATGL) and hormone sensitive lipase (HSL) promote lipolysis (hydrolysis of triacylglycerol to free fatty acids and glycerol) in WAT [56]. Overexpression of ATGL and HSL in the WAT of cancer cachexia patients is well documented and correlates with falling BMI [57]. Suppression of either HSL or ATGL expression in mice preserved not only WAT, it also protected against skeletal muscle loss, suggesting important cross talk between the AT and muscle in cancer cachexia [57,58] (Figure 3 and Figure 4).

## 8. Breakdown of Muscle Fibers

Whereas adipose tissue mainly contributes to weight loss in starvation, both skeletal muscle and adipose tissue mass are severely depleted in cancer cachexia [60]. A number of tumor/host-associated factors contribute to cancer cachexia associated loss of muscle fibers. Both protein catabolism and suppression of muscle growth participate in the muscle atrophy process. These are mediated by pro-inflammatory cytokines including TNF-α, IL-1, IL-6, IFN-γ, and proteolysis inducing factor (PIF) as well as other circulating TGF-β related molecules such as myostatin and Activin A [61]. Pro-inflammatory cytokines exert their actions through their cognate receptors, initiating intracellular signaling cascades including the p38 mitogen-activated protein kinases (MAPK) and NF-κB pathways [62].

TGF-β signaling via SMAD transcription factors results in the induction of the ubiquitin ligases muscle atrophy F-box protein (MAFBX or atrogin-1) and muscle RING finger-containing protein 1 (MuRF-1). The TGF-β superfamily members myostatin and Activin A share the membrane Activin type II receptor B (ActRIIB) [63]. Thus, myostatin is a key suppressor of muscle anabolism [64,65]. Myostatin activates the Smad/atrogin-1/MuRF-1 signaling cascade, and suppresses PI3K/AKT activity. Normally, PI3K/AKT activation results in phosphorylation of the FoxO family of transcription factors. Myostatin relieves the PI3K/AKT mediated inhibition of FoxO, allowing them to stimulate autophagy, promote expression of atrogin-1/MuRF-1, and upregulate other mediators of muscle catabolism [62]. The mentioned catabolic pathways including FoxO and Smad2/3 inhibit the IGF-1 signaling axis, one of the most well characterized anabolic signaling cascades [62]. Furthermore, Activin A, a member of the TGF-β superfamily that is produced by both tumor and immune cells, promotes atrophy in myotubes in murine models [61,66]. Activin A concentration is elevated in cancer cachexia in humans and correlates with weight loss [66]. Pharmacological blockade of the ActRIIB pathway with anti-ActRII antibodies (e.g., bimagrumab) showed promising results in animal models and human trials, not only preventing further muscle wasting, but reversing cancer-induced loss of skeletal muscle and cardiac atrophy [67,68]. 

The loss of fat mass in cancer cachexia is associated with elevated levels of intramyocellular lipid droplets. This is a sign of defective fatty acid (FA) utilization and mitochondrial β-oxidation [69], with important consequences for muscle performance. Apart from decreased skeletal muscle oxidative capacity and ATP production, other contributing factors in muscle attrition include oxidatively modified mitochondrial proteins, disrupted muscle protein synthesis, and increased mitochondrial membrane permeability [12]. The resulting decrease in skeletal muscle density (SMD) and the extent of sarcopenia can be measured by CT scanning (e.g., psoas muscle volume). Sarcopenia is predictive of poor prognosis in cancer patients and correlates with the toxicity of chemotherapy and increased surgical morbidity [70,71,72,73]. Sarcopenia is an independent prognostic indicator of poor overall survival in patients with advanced cancer stage [74]. Mechanisms underlying muscle wasting in cancer CAS are illustrated in Figure 4.

A major acceleration in the attrition of muscle and adipose tissue is observed in the final stage of disease in patients with metastatic colorectal cancer during the 100 days preceding death. This is concurrent with uncontrolled activity of high metabolic rate tissues including the liver and treatment-resistant metastatic disease [75].

## 9. Multifactorial Etiology of Cachexia-Anorexia Syndrome in Peritoneal Metastasis

**Figure 5:**
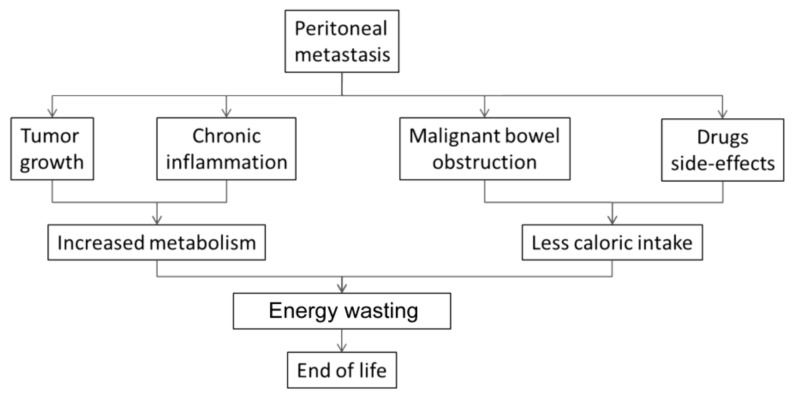
Cachexia-Anorexia Syndrome (CAS) in PM patients. In cancer CAS, energy metabolism is increased by tumor growth and chronic inflammation. Host caloric intake is impaired by tumor invasion of the bowel, ascites, side-effects of drugs, and appetite suppression.

The metabolic derangement observed in CAS is caused by increased metabolism and decreased caloric intake. Both tumor growth and systemic inflammatory response lead to considerable energy wastage. Anorexia results in poor caloric intake, and is particularly pronounced in PM patients due to bowel tumor invasion, abdominal pain, and ascites. The multifactorial etiology of CAS in PM patients is illustrated in Figure 5. 

In PM patients, the following specific components contribute to the development of CAS:

### 9.1. Bowel Invasion

Tumor spreading within the peritoneal cavity causes progressive intestinal dysfunction by infiltration of the bowel wall. Patients report ill-defined abdominal pain, nausea, and anorexia, which in turn impair adequate oral nutritional intake [14]. Contrast-enhanced (intraluminal and intravenous) abdominal CT-scan can show centimetric peritoneal nodes, transition points and high grade obstruction [76]. However, CT-scan has only limited sensitivity for small-volumetric peritoneal disease and underestimates diffuse small bowel involvement, particularly in patients receiving systemic chemotherapy [77]. In our experience, patency of the small bowel is still present in a substantial proportion of PM patients presenting with nausea and anorexia, as demonstrated by small bowel series. However, muscular and serosal layers of the bowel are involved, resulting in progressive and life-threatening intestinal failure [8].

### 9.2. Drug-Related Side Effects

Opioid receptors are expressed throughout the gastrointestinal tract. When stimulated by exogenous opioids, there are decreases in motility, secretion, and absorption of fluids, and increased sphincter function (pylorus/anal) [78]. PM patients are commonly prescribed morphine derivatives because of visceral and abdominal pain. Opioids inhibit gastrointestinal and colonic transit by initiating non-propulsive contraction: the “opioid-induced bowel dysfunction syndrome” [79].

### 9.3. Omental Metastasis

Cancer cells shed into the peritoneal cavity frequently implant onto the omentum. This is mediated by chemokine homing from omental adipokine release and tumor PGK-1 expression. Omental adipocytes can provide alternative energy substrates to glucose, which enables shed cancer cells to resist anoikis. Stored triglycerides in omental adipocytes undergo lipolysis to provide free fatty acids (FFA) and glycerol. Beta-oxidation of these FFAs can be used by cancer cells to produce acetyl-CoA for the Kreb’s cycle or for cell membrane production (e.g., phospholipids). Thus, fat stores can be utilized by PM for further growth, contributing to CAS in the patient [24].

### 9.4. Systemic Palliative Chemotherapy 

Systemic palliative chemotherapy, which is the standard of care in PM patients, induces gastrointestinal symptoms in a substantial proportion of patients, with 19–58% of oncology patients experiencing chemotherapy-induced nausea (CIN) [80]. Systemic cytotoxic chemotherapy can affect all rapidly dividing cells including epithelial cells in the gastrointestinal mucosa [10]. This results in PM patients experiencing a cluster of gastrointestinal side effects such as lack of appetite, mucositis, mouth ulcers, difficulty eating or swallowing, nausea, vomiting, diarrhea, abdominal pain, and malabsorption [81]. Thus, the risk-benefit ratio of palliative, combination systemic chemotherapy should be carefully evaluated in individual PM patients, particularly in salvage treatments for chemo-resistant tumors. 

## 10. Therapy of CAS in PM Patients

The etiology of CAS in PM is multifactorial. Therapy should ideally target these multiple factors, in particular counteracting the anorexia, systemic inflammation, wasting, and hypermetabolism suffered by the cancer patient. The multimodal treatment approach in CAS includes drug therapies, nutritional support, and physical exercise. Numerous intervention studies in CAS include the use of immunonutrition, immunotherapy (pembroluzimab), L-carnitine, Th1 cytokine blockade (infliximab, etanercept, clazakizumab, MABp1), synthetic ghrelin receptor agonists (anamorelin), NSAIDS (celecoxib), angiotensin II inhibitors (telmisartan), beta-adrenergic antagonists (espindolol, propranolol), β2-adrenergic agonists (formoterol), selective androgen receptor modulators (SARMS, e.g., enobosarm), and anti-myostatin peptides [12,51,82]. Despite completing phase III trials, anamorelin, enobosarm, and MABp1 have not as yet gained either US FDA or European Medical Agency (EMA) regulatory approval for cancer CAS treatment, due to toxicity or a lack of meaningful clinical response in single agent drugs [83,84]. 

To our knowledge, there are no specific and broadly accepted guidelines for treating CAS in PM patients. PM patients report disabling symptoms and complications that dramatically impact their quality of life. There are several endoscopic or surgical procedures to palliate symptoms such as bowel dysfunction, nausea, abdominal pain, and anorexia in these patients.

Bowel dysfunction is a combined surgical and medical challenge. In PM patients, it can be difficult to distinguish between bowel dysfunction, incomplete, and complete bowel obstruction. In the presence of localized bowel obstruction, endoscopic stenting is the preferred option but is only feasible in the proximal or distal bowel, for example, for relief of a gastric outlet obstruction or a sigmoid colon stenosis. In the presence of diffuse bowel involvement, indications for laparotomy and surgical bypass must be carefully considered, placement of an ostomy being one palliative option with a better risk/benefit ratio for some patients. Placement of a gastrostomy tube for decompression is another option for palliation of gastric outlet, duodenal and non-operable bowel obstruction, or gastrointestinal dysmotility [8]. Pharmaceutical therapy of bowel obstruction includes anti-emetics, corticosteroids, octreotide, and anti-cholinergic agents. A combination of analgesics, anti-emetics, and anti-cholinergics with or without anti-secretory agents can successfully improve symptom control in patients with irreversible bowel obstruction [85].

In PM, treating abdominal pain with opioids often results in nausea, severe constipation, and further impairment of bowel function. Thus, as long as possible, analgesia in PM patients should be achieved with other agents than opioids. In anecdotal cases, cannabinoids have been used for alleviating symptoms of CAS in PM. The endocannabinoid system (ECS) is a widely distributed transmitter system that peripherally and centrally controls gut function and endorphin release. It is an important physiological regulator of gastrointestinal motility and involved in the control of nausea, vomiting, and visceral sensation [86]. Cannabis has been used for the treatment of CAS, however, the adverse psychotropic side effects of THC have often limited its use. Furthermore, two randomized trials in cancer CAS comparing cannabinoids to megestrol acetate [87] and placebo [88] failed to show clinical benefit [51]. More selective or peripherally acting cannabinoids are under development and may become clinically available for the control of abdominal pain, nausea, anorexia, and vomiting [89] in PM patients. 

Finally, it was recently reported that an innovative drug delivery platform, pressurized intraperitoneal aerosol chemotherapy (PIPAC) can stabilize nutritional status and improve abdominal symptoms in a substantial proportion of patients with PM of ovarian origin [22]. Application of low-dose cisplatin and doxorubicin as pressurized aerosols during laparoscopy, repeated at 6-week intervals, stabilized or improved CAS in 85% patients with follow-up data. These promising results need to be confirmed in controlled prospective studies. 

## 11. Conclusions and Outlook

The challenges raised by CAS in patients with PM are considerable. Little attention is often paid to this topic, since PM is still perceived as a terminal condition by many physicians. However, with the emergence of novel multimodal therapies combining cytoreductive surgery, novel chemotherapeutic drugs, immunotherapy, and local interventions, an increasing number of long-term survivors have been reported in the international literature. In parallel, knowledge of the metabolic dysfunction associated with CAS in PM is growing, a number of potential drug targets have been identified, and several drugs are under clinical development. Thus, it is expected that novel drugs targeting metabolic enzymes in combination with innovative drug delivery systems may improve the current standard of care, and contribute to improved control of the negative energy balance, catabolic metabolism, and symptoms of CAS in patients with PM. 

## Figures and Tables

**Table 1:** Characteristics of starvation vs. cancer cachexia anorexia syndrome (CAS) (modified from [6]).

Starvation Response	Cancer Cachexia Anorexia Syndrome (CAS)
Physiologic response to low energy intake	Pathologic response (inflammation, cytokines, hormonal changes)
Preserved appetite and ghrelin response	Ghrelin resistance and loss of appetite
Decreased REE	Increased REE
Use of fat > muscle stores	Use of both fat and muscle for energy production
Protein catabolism reduced	Increased protein turnover
Reversible	Difficult to reverse

## References

[1] Dev R. (2019). Measuring cachexia-diagnostic criteria. Ann. Palliat. Med..

[2] Fearon K., Strasser F., Anker S.D., Bosaeus I., Bruera E., Fainsinger R.L., Jatoi A., Loprinzi C., MacDonald N., Mantovani G. (2011). Definition and classification of cancer cachexia: An international consensus. Lancet Oncol..

[3] Nordhausen K., Solass W., Demtroeder C., Tempfer C.B., Reymond M. (2016). Cachexia-anorexia syndrome in patients with peritoneal metastasis: An observational study. Pleura Peritoneum.

[4] Suzuki H., Asakawa A., Amitani H., Nakamura N., Inui A. (2013). Cancer cachexia--pathophysiology and management. J. Gastroenterol..

[5] Walsh D., Donnelly S., Rybicki L. (2000). The symptoms of advanced cancer: Relationship to age, gender, and performance status in 1000 patients. Support. Care Cancer.

[6] Childs D.S., Jatoi A. (2019). A hunger for hunger: A review of palliative therapies for cancer-associated anorexia. Ann. Palliat. Med..

[7] Bray F., Ferlay J., Soerjomataram I., Siegel R.L., Torre L.A., Jemal A. (2018). Global cancer statistics 2018: GLOBOCAN estimates of incidence and mortality worldwide for 36 cancers in 185 countries. CA Cancer J. Clin..

[8] Lambert L.A., Wiseman J. (2018). Palliative Management of Peritoneal Metastases. Ann. Surg. Oncol.

[9] Klein C., Stiel S., Bukki J., Ostgathe C. (2012). Pharmacological treatment of malignant bowel obstruction in severely ill and dying patients: A systematic literature review. Schmerz.

[10] Norman K., Pichard C., Lochs H., Pirlich M. (2008). Prognostic impact of disease-related malnutrition. Clin. Nutr..

[11] Maeda O., Ando T., Ishiguro K., Watanabe O., Miyahara R., Nakamura M., Funasaka K., Kazuhiro F., Ando Y., Goto H. (2014). Safety of repeated cell-free and concentrated ascites reinfusion therapy for malignant ascites from gastrointestinal cancer. Mol. Clin. Oncol..

[12] Argiles J.M., Busquets S., Stemmler B., Lopez-Soriano F.J. (2014). Cancer cachexia: Understanding the molecular basis. Nat. Rev. Cancer.

[13] Evans W.K., Makuch R., Clamon G.H., Feld R., Weiner R.S., Moran E., Blum R., Shepherd F.A., Jeejeebhoy K.N., DeWys W.D. (1985). Limited impact of total parenteral nutrition on nutritional status during treatment for small cell lung cancer. Cancer Res..

[14] Rigaud D., Hassid J., Meulemans A., Poupard A.T., Boulier A. (2000). A paradoxical increase in resting energy expenditure in malnourished patients near death: The king penguin syndrome. Am. J. Clin. Nutr..

[15] Secor S.M., Carey H.V. (2016). Integrative Physiology of Fasting. Compr. Physiol..

[16] Soeters M.R., Soeters P.B., Schooneman M.G., Houten S.M., Romijn J.A. (2012). Adaptive reciprocity of lipid and glucose metabolism in human short-term starvation. Am. J. Physiol. Endocrinol. Metab..

[17] Belkhou R., Bechet D., Cherel Y., Galluser M., Ferrara M., le Maho Y. (1994). Effect of fasting and thyroidectomy on cysteine proteinase activities in liver and muscle. Biochim. Biophys. Acta.

[18] Goodman M.N., Larsen P.R., Kaplan M.M., Aoki T.T., Young V.R., Ruderman N.B. (1980). Starvation in the rat. II. Effect of age and obesity on protein sparing and fuel metabolism. Am. J. Physiol.

[19] McCue M.D., Terblanche J.S., Benoit J.B. (2017). Learning to starve: Impacts of food limitation beyond the stress period. J. Exp. Biol..

[20] Friesen D.E., Baracos V.E., Tuszynski J.A. (2015). Modeling the energetic cost of cancer as a result of altered energy metabolism: Implications for cachexia. Biol. Med. Model..

[21] Cao D.X., Wu G.H., Zhang B., Quan Y.J., Wei J., Jin H., Jiang Y., Yang Z.A. (2010). Resting energy expenditure and body composition in patients with newly detected cancer. Clin. Nutr..

[22] Hilal Z., Rezniczek G.A., Klenke R., Dogan A., Tempfer C.B. (2017). Nutritional status, cachexia, and anorexia in women with peritoneal metastasis and intraperitoneal chemotherapy: A longitudinal analysis. J. Gynecol. Oncol..

[23] Steinert R., Hantschick M., Vieth M., Gastinger I., Kuhnel F., Lippert H., Reymond M.A. (2008). Influence of subclinical tumor spreading on survival after curative surgery for colorectal cancer. Arch. Surg..

[24] Wilson R.B., Solass W., Archid R., Weinreich F.J., Konigsrainer A., Reymond M.A. (2019). Resistance to anoikis in transcoelomic shedding: The role of glycolytic enzymes. Pleura Peritoneum.

[25] Wilde L., Roche M., Domingo-Vidal M., Tanson K., Philp N., Curry J., Martinez-Outschoorn U. (2017). Metabolic coupling and the Reverse Warburg Effect in cancer: Implications for novel biomarker and anticancer agent development. Semin. Oncol..

[26] Kim J.M., Sung M.K. (2016). The Efficacy of Oral Nutritional Intervention in Malnourished Cancer Patients: A Systemic Review. Clin. Nutr. Res..

[27] Porporato P.E. (2016). Understanding cachexia as a cancer metabolism syndrome. Oncogenesis.

[28] Warburg O., Wind F., Negelein E. (1927). The Metabolism of Tumors in the Body. J. Gen. Physiol..

[29] Wilson R.B. (2018). Hypoxia, cytokines and stromal recruitment: Parallels between pathophysiology of encapsulating peritoneal sclerosis, endometriosis and peritoneal metastasis. Pleura Peritoneum.

[30] Rempel A., Mathupala S.P., Griffin C.A., Hawkins A.L., Pedersen P.L. (1996). Glucose catabolism in cancer cells: Amplification of the gene encoding type II hexokinase. Cancer Res..

[31] Denko N.C. (2008). Hypoxia, HIF1 and glucose metabolism in the solid tumour. Nat. Rev. Cancer.

[32] Kluza J., Corazao-Rozas P., Touil Y., Jendoubi M., Maire C., Guerreschi P., Jonneaux A., Ballot C., Balayssac S., Valable S. (2012). Inactivation of the HIF-1alpha/PDK3 signaling axis drives melanoma toward mitochondrial oxidative metabolism and potentiates the therapeutic activity of pro-oxidants. Cancer Res..

[33] Avagliano A., Granato G., Ruocco M.R., Romano V., Belviso I., Carfora A., Montagnani S., Arcucci A. (2018). Metabolic Reprogramming of Cancer Associated Fibroblasts: The Slavery of Stromal Fibroblasts. Biomed. Res. Int..

[34] Martinez-Outschoorn U.E., Lin Z., Trimmer C., Flomenberg N., Wang C., Pavlides S., Pestell R.G., Howell A., Sotgia F., Lisanti M.P. (2011). Cancer cells metabolically “fertilize” the tumor microenvironment with hydrogen peroxide, driving the Warburg effect: Implications for PET imaging of human tumors. Cell Cycle.

[35] Wallace D.C. (2012). Mitochondria and cancer. Nat. Rev. Cancer.

[36] Martinez-Outschoorn U.E., Pavlides S., Howell A., Pestell R.G., Tanowitz H.B., Sotgia F., Lisanti M.P. (2011). Stromal-epithelial metabolic coupling in cancer: Integrating autophagy and metabolism in the tumor microenvironment. Int. J. Biochem. Cell Biol..

[37] Argiles J.M., Busquets S., Stemmler B., Lopez-Soriano F.J. (2015). Cachexia and sarcopenia: Mechanisms and potential targets for intervention. Curr. Opin. Pharmacol..

[38] Capparelli C., Whitaker-Menezes D., Guido C., Balliet R., Pestell T.G., Howell A., Sneddon S., Pestell R.G., Martinez-Outschoorn U., Lisanti M.P. (2012). CTGF drives autophagy, glycolysis and senescence in cancer-associated fibroblasts via HIF1 activation, metabolically promoting tumor growth. Cell Cycle.

[39] Martinez-Outschoorn U.E., Lisanti M.P., Sotgia F. (2014). Catabolic cancer-associated fibroblasts transfer energy and biomass to anabolic cancer cells, fueling tumor growth. Semin. Cancer Biol..

[40] Roy A., Bera S. (2016). CAF cellular glycolysis: Linking cancer cells with the microenvironment. Tumour. Biol..

[41] Shangguan C., Gan G., Zhang J., Wu J., Miao Y., Zhang M., Li B., Mi J. (2018). Cancer-associated fibroblasts enhance tumor (18)F-FDG uptake and contribute to the intratumor heterogeneity of PET-CT. Theranostics.

[42] Khan M.I., Rath S., Adhami V.M., Mukhtar H. (2018). Hypoxia driven glycation: Mechanisms and therapeutic opportunities. Semin. Cancer Biol..

[43] Uribarri J., del Castillo M.D., de la Maza M.P., Filip R., Gugliucci A., Luevano-Contreras C., Macias-Cervantes M.H., Markowicz Bastos D.H., Medrano A., Menini T. (2015). Dietary advanced glycation end products and their role in health and disease. Adv. Nutr..

[44] Kang R., Tang D., Livesey K.M., Schapiro N.E., Lotze M.T., Zeh H.J. (2011). The Receptor for Advanced Glycation End-products (RAGE) protects pancreatic tumor cells against oxidative injury. Antioxid. Redox Signal..

[45] Riuzzi F., Sorci G., Sagheddu R., Chiappalupi S., Salvadori L., Donato R. (2018). RAGE in the pathophysiology of skeletal muscle. J. Cachexia Sarcopenia Muscle.

[46] Sessa L., Gatti E., Zeni F., Antonelli A., Catucci A., Koch M., Pompilio G., Fritz G., Raucci A., Bianchi M.E. (2014). The receptor for advanced glycation end-products (RAGE) is only present in mammals, and belongs to a family of cell adhesion molecules (CAMs). PLoS ONE.

[47] Lu S., Wang Y. (2018). Nonmetabolic functions of metabolic enzymes in cancer development. Cancer Commun..

[48] Tazzari M., Brich S., Tuccitto A., Bozzi F., Beretta V., Spagnuolo R.D., Negri T., Stacchiotti S., Deraco M., Baratti D. (2018). Complex Immune Contextures Characterise Malignant Peritoneal Mesothelioma: Loss of Adaptive Immunological Signature in the More Aggressive Histological Types. J. Immunol. Res..

[49] Shrotriya S., Walsh D., Nowacki A.S., Lorton C., Aktas A., Hullihen B., Benanni-Baiti N., Hauser K., Ayvaz S., Estfan B. (2018). Serum C-reactive protein is an important and powerful prognostic biomarker in most adult solid tumors. PLoS ONE.

[50] Banks W.A. (2001). Anorectic effects of circulating cytokines: Role of the vascular blood-brain barrier. Nutrition.

[51] Aoyagi T., Terracina K.P., Raza A., Matsubara H., Takabe K. (2015). Cancer cachexia, mechanism and treatment. World J. Gastrointest. Oncol..

[52] Schcolnik-Cabrera A., Chavez-Blanco A., Dominguez-Gomez G., Duenas-Gonzalez A. (2017). Understanding tumor anabolism and patient catabolism in cancer-associated cachexia. Am. J. Cancer Res..

[53] Coss C.C., Clinton S.K., Phelps M.A. (2018). Cachectic Cancer Patients: Immune to Checkpoint Inhibitor Therapy?. Clin. Cancer Res. Off. J. Am. Assoc. Cancer Res..

[54] Mace T.A., Shakya R., Pitarresi J.R., Swanson B., McQuinn C.W., Loftus S., Nordquist E., Cruz-Monserrate Z., Yu L., Young G. (2018). IL-6 and PD-L1 antibody blockade combination therapy reduces tumour progression in murine models of pancreatic cancer. Gut.

[55] Ebadi M., Mazurak V.C. (2014). Evidence and mechanisms of fat depletion in cancer. Nutrients.

[56] Tsoli M., Swarbrick M.M., Robertson G.R. (2016). Lipolytic and thermogenic depletion of adipose tissue in cancer cachexia. Seminars in Cell & Developmental Biology.

[57] Dalal S. (2019). Lipid metabolism in cancer cachexia. Ann. Palliat. Med..

[58] Das S.K., Eder S., Schauer S., Diwoky C., Temmel H., Guertl B., Gorkiewicz G., Tamilarasan K.P., Kumari P., Trauner M. (2011). Adipose triglyceride lipase contributes to cancer-associated cachexia. Science.

[59] Argiles J.M., Lopez-Soriano F.J., Busquets S. (2019). Mediators of cachexia in cancer patients. Nutrition.

[60] Moley J.F., Aamodt R., Rumble W., Kaye W., Norton J.A. (1987). Body cell mass in cancer-bearing and anorexic patients. J. Parenter Enter. Nutr..

[61] Baracos V.E., Martin L., Korc M., Guttridge D.C., Fearon K.C.H. (2018). Cancer-associated cachexia. Nat. Rev. Dis. Primers..

[62] Carr R.M., Enriquez-Hesles E., Olson R.L., Jatoi A., Doles J., Fernandez-Zapico M.E. (2017). Epigenetics of cancer-associated muscle catabolism. Epigenomics.

[63] Xia Y., Schneyer A.L. (2009). The biology of activin: Recent advances in structure, regulation and function. J. Endocrinol..

[64] McPherron A.C. (2010). Metabolic Functions of Myostatin and Gdf11. Immunol. Endocr. Metab. Agents Med. Chem..

[65] McPherron A.C., Lawler A.M., Lee S.J. (1997). Regulation of skeletal muscle mass in mice by a new TGF-beta superfamily member. Nature.

[66] Loumaye A., de Barsy M., Nachit M., Lause P., Frateur L., van Maanen A., Trefois P., Gruson D., Thissen J.P. (2015). Role of Activin A and myostatin in human cancer cachexia. J. Clin. Endocrinol. Metab..

[67] Busquets S., Toledo M., Orpi M., Massa D., Porta M., Capdevila E., Padilla N., Frailis V., Lopez-Soriano F.J., Han H.Q. (2012). Myostatin blockage using actRIIB antagonism in mice bearing the Lewis lung carcinoma results in the improvement of muscle wasting and physical performance. J. Cachexia Sarcopenia Muscle.

[68] Zhou X., Wang J.L., Lu J., Song Y., Kwak K.S., Jiao Q., Rosenfeld R., Chen Q., Boone T., Simonet W.S. (2010). Reversal of cancer cachexia and muscle wasting by ActRIIB antagonism leads to prolonged survival. Cell.

[69] Stephens N.A., Skipworth R.J., Macdonald A.J., Greig C.A., Ross J.A., Fearon K.C. (2011). Intramyocellular lipid droplets increase with progression of cachexia in cancer patients. J. Cachexia Sarcopenia Muscle.

[70] Antoun S., Lanoy E., Iacovelli R., Albiges-Sauvin L., Loriot Y., Merad-Taoufik M., Fizazi K., di Palma M., Baracos V.E., Escudier B. (2013). Skeletal muscle density predicts prognosis in patients with metastatic renal cell carcinoma treated with targeted therapies. Cancer.

[71] Aubrey J., Esfandiari N., Baracos V.E., Buteau F.A., Frenette J., Putman C.T., Mazurak V.C. (2014). Measurement of skeletal muscle radiation attenuation and basis of its biological variation. Acta Physiol..

[72] Goodpaster B.H., Kelley D.E., Thaete F.L., He J., Ross R. (2000). Skeletal muscle attenuation determined by computed tomography is associated with skeletal muscle lipid content. J. Appl. Physiol..

[73] Prado C.M., Baracos V.E., McCargar L.J., Reiman T., Mourtzakis M., Tonkin K., Mackey J.R., Koski S., Pituskin E., Sawyer M.B. (2009). Sarcopenia as a determinant of chemotherapy toxicity and time to tumor progression in metastatic breast cancer patients receiving capecitabine treatment. Clin. Cancer Res. Off. J. Am. Assoc. Cancer Res..

[74] Lee J.S., Kim Y.S., Kim E.Y., Jin W. (2018). Prognostic significance of CT-determined sarcopenia in patients with advanced gastric cancer. PLoS ONE.

[75] Lieffers J.R., Mourtzakis M., Hall K.D., McCargar L.J., Prado C.M., Baracos V.E. (2009). A viscerally driven cachexia syndrome in patients with advanced colorectal cancer: Contributions of organ and tumor mass to whole-body energy demands. Am. J. Clin. Nutr..

[76] Laghi A., Bellini D., Rengo M., Accarpio F., Caruso D., Biacchi D., Di Giorgio A., Sammartino P. (2017). Diagnostic performance of computed tomography and magnetic resonance imaging for detecting peritoneal metastases: Systematic review and meta-analysis. Radiol. Med..

[77] Goswami G., Kammar P., Mangal R., Shaikh S., Patel M.D., Bhatt A. (2019). Accuracy of CT Scan in Predicting the Surgical PCI in Patients Undergoing Cytoreductive Surgery with/without HIPEC-a Prospective Single Institution Study. Indian J. Surg. Oncol..

[78] Drewes A.M., Munkholm P., Simren M., Breivik H., Kongsgaard U.E., Hatlebakk J.G., Agreus L., Friedrichsen M., Christrup L.L. (2016). Definition, diagnosis and treatment strategies for opioid-induced bowel dysfunction-Recommendations of the Nordic Working Group. Scand. J. Pain.

[79] Muller-Lissner S., Bassotti G., Coffin B., Drewes A.M., Breivik H., Eisenberg E., Emmanuel A., Laroche F., Meissner W., Morlion B. (2017). Opioid-Induced Constipation and Bowel Dysfunction: A Clinical Guideline. Pain Med..

[80] Singh K., Kober K.M., Paul S.M., Hammer M., Wright F., Conley Y.P., Levine J.D., Miaskowski C. (2019). Gastrointestinal symptoms are associated with trajectories of chemotherapy-induced nausea. Support. Care Cancer.

[81] Cherwin C.H. (2012). Gastrointestinal symptom representation in cancer symptom clusters: A synthesis of the literature. Oncol. Nurs. Forum.

[82] Argiles J.M., Lopez-Soriano F.J., Stemmler B., Busquets S. (2019). Therapeutic strategies against cancer cachexia. Eur J. Transl. Myol..

[83] Dev R., Wong A., Hui D., Bruera E. (2017). The Evolving Approach to Management of Cancer Cachexia. Oncology.

[84] Garcia J.M. (2017). What is next after anamorelin?. Curr. Opin. Support. Palliat. Care.

[85] O’Connor B., Creedon B. (2011). Pharmacological treatment of bowel obstruction in cancer patients. Expert Opin. Pharmacother..

[86] Sharkey K.A., Wiley J.W. (2016). The Role of the Endocannabinoid System in the Brain-Gut Axis. Gastroenterology.

[87] Jatoi A., Windschitl H.E., Loprinzi C.L., Sloan J.A., Dakhil S.R., Mailliard J.A., Pundaleeka S., Kardinal C.G., Fitch T.R., Krook J.E. (2002). Dronabinol versus megestrol acetate versus combination therapy for cancer-associated anorexia: A North Central Cancer Treatment Group study. J. Clin. Oncol..

[88] Cannabis In Cachexia Study G., Strasser F., Luftner D., Possinger K., Ernst G., Ruhstaller T., Meissner W., Ko Y.D., Schnelle M., Reif M. (2006). Comparison of orally administered cannabis extract and delta-9-tetrahydrocannabinol in treating patients with cancer-related anorexia-cachexia syndrome: A multicenter, phase III, randomized, double-blind, placebo-controlled clinical trial from the Cannabis-In-Cachexia-Study-Group. J. Clin. Oncol..

[89] Malik Z., Baik D., Schey R. (2015). The role of cannabinoids in regulation of nausea and vomiting, and visceral pain. Curr. Gastroenterol. Rep..

